# Assessment of circulating tumor cells and serum markers for progression-free survival prediction in metastatic breast cancer: a prospective observational study

**DOI:** 10.1186/bcr3114

**Published:** 2012-02-13

**Authors:** François-Clément Bidard, David Hajage, Thomas Bachelot, Suzette Delaloge, Etienne Brain, Mario Campone, Paul Cottu, Philippe Beuzeboc, Emilie Rolland, Claire Mathiot, Jean-Yves Pierga

**Affiliations:** 1 Department of Medical Oncology, Institut Curie, 26 rue d'Ulm, 75005 Paris, France; 2 Université Paris Descartes, 12 rue de l'école de Médecine, 75006 Paris, France; 3 Department of Biostatistics, Institut Curie, 26 rue d'Ulm, 75005 Paris, France; 4 Department of Medical Oncology, Centre Léon Bérard, 28 rue Laënnec, 69008 Lyon, France; 5 Department of Medical Oncology, Institut Gustave Roussy, 114 rue Vaillant, 94800 Villejuif, France; 6 Department of Medical Oncology, Institut Curie, Hôpital René Huguenin, 35 rue Dailly, 92210 Saint Cloud, France; 7 Department of Medical Oncology, Institut de Cancérologie de l'Ouest, Bd Monod, 44800 Saint Herblain, France; 8 Hematology laboratory, Institut Curie, 26 rue d'Ulm, 75005 Paris, France

## Abstract

**Introduction:**

Circulating tumor cells (CTC) have been recently proposed as a new dynamic blood marker whose positivity at baseline is a prognostic factor and whose changes under treatment are correlated with progression-free survival (PFS) in metastatic breast cancer patients. However, serum marker levels are also used for the same purpose, and no clear comparison has been reported to date.

**Methods:**

The IC 2006-04 enrolled prospectively 267 metastatic breast cancer patients treated by first line chemotherapy and confirmed that CTC levels are an independent prognostic factor for PFS and overall survival (OS). A secondary pre-planned endpoint was to compare prospectively the positivity rates and the value of CTC (CellSearch^®^), of serum tumor markers (carcinoembryonic antigen (CEA), cancer antigen 15.3 (CA 15-3), CYFRA 21-1), and of serum non-tumor markers (lactate deshydrogenase (LDH), alkaline phosphatase (ALP)) at baseline and under treatment for PFS prediction, independently from the other known prognostic factors, using univariate analyses and concordance indexes.

**Results:**

A total of 90% of the patients had at least one elevated blood marker. Blood markers were correlated with poor performance status, high number of metastatic sites and with each other. In particular, CYFRA 21-1, a marker usually used in lung cancer, was elevated in 65% of patients. A total of 86% of patients had either CA 15-3 and/or CYFRA 21-1 elevated at baseline. Each serum marker was associated, when elevated at baseline, with a significantly shorter PFS. Serum marker changes during treatment, assessed either between baseline and week 3 or between baseline and weeks 6 to 9, were significantly associated with PFS, as reported for CTC. Concordance indexes comparison showed no clear superiority of any of the serum marker or CTC for PFS prediction.

**Conclusions:**

For the purpose of PFS prediction by measuring blood marker changes during treatment, currently available blood-derived markers (CTC and serum markers) had globally similar performances. Besides CEA and CA 15-3, CYFRA 21-1 is commonly elevated in metastatic breast cancer and has a strong prognostic value.

## Introduction

Several serum markers have been developed in different types of cancer as tools for non-invasive assessment of the tumor burden, mostly in metastatic patients. Quantitative variations of serum markers are, therefore, often used in several cancer types as noninvasive tools to assess treatment efficiency in metastatic patients. However, the use of serum tumor markers faces several issues and unanswered questions: their specificity and sensitivity are considered as low and no clear consensus exists on what threshold and/or variation should be considered clinically significant and which serum marker to follow.

In breast cancer, the commonly used serum markers are carcinoembryonic antigen (CEA), cancer antigen 27.29 (CA 27.29), and cancer antigen 15.3 (CA 15-3) [1]. CEA is a cell surface glycoprotein involved in cell adhesion, normally not present in the blood of healthy adults. CA 27.29, mostly used in North America, and CA 15-3, mostly used in Europe, correspond to two different epitopes of the same protein, MUC1, which is also a cell surface glycoprotein involved in cell adhesion. CYFRA 21-1, which is a commonly used serum marker in lung cancer [2], consists of cytokeratin-19 fragments which are specifically recognized by two monoclonal antibodies originally derived after injecting breast cancer cells (MCF7 cell line) into mice [3]. Small published reports, together with an empiric background at the Institut Curie, suggested that CYFRA 21-1 is commonly elevated and may be used for the management of metastatic breast cancers [4,5]. The 2007 American Society of Clinical Oncology update on tumor markers in breast cancer [6] reported that for monitoring patients with metastatic disease during therapy, CA 27.29 or CA 15-3 can be used in conjunction with other monitoring tools, such as tumor response radiological assessment.

In 2004, circulating tumor cells (CTC) detection in blood by the CellSearch^® ^system (Veridex, Raritan NJ, USA) was reported to be a prognostic marker in a study by Cristofanilli *et al*. [7]; moreover, changes in CTC count after one cycle of chemotherapy were associated with progression-free survival (PFS) [8]. On the basis of this first study, the FDA cleared the use of the CellSearch^® ^system as a tool for monitoring chemotherapy in metastatic breast cancer patients, although no comparison with serum marker was initially reported. The prospective multicentric IC 2006-04 study was initiated in 2006 as a confirmatory study of the Cristofanilli's study, with the correlation of CTC changes and survival as the primary objective. We have recently reported this confirmatory objective of the study, which was clearly reached: CTC changes are a strong prognostic factor for both PFS and overall survival (OS) [9]. We report here, for the first time, the comparison of CTC with different serum tumor markers (CEA, CA 15-3, CYFRA 21-1) and non-tumor markers (lactate deshydrogenase LDH, alkaline phosphatase ALP), which was a prospectively planned secondary objective of the IC 2006-04 study.

## Materials and methods

This prospective study was approved by the national ethics board, identified as DGS 2006-A00523-48 (France) and NCT00898014 (USA) and was conducted in five different French comprehensive cancer centers.

### Patients and treatment

The main eligibility requirements for this study included the patient's written informed consent, metastatic breast cancer, patients entering first-line chemotherapy (chosen by clinicians) with or without targeted therapy, life expectancy of at least three months, and measurable or evaluable disease. Clinical evaluations were conducted as usual, but were mandatory at inclusion (before cycle 1, that is, week 0) and at the first radiological evaluation before cycle 3 or 4 (C3/4, that is, weeks 6 to 9). Radiological evaluations (RECIST [10]) were mandatory at inclusion and before C3/4. The following blood tests were obtained at inclusion, before cycle 2 (C2), C3/4, and at progression: complete blood count, liver function (including LDH and ALP), serum calcium and serum markers: CEA, CA 15-3 and CYFRA 21-1. These blood tests were processed locally and disclosed to clinicians. CTC were analyzed at four different time-points in an experienced laboratory (Institut Curie, Paris): at inclusion (before starting treatment), before C2, before C3/4 and at disease progression (not shown). CTC counts were not disclosed to clinicians. Technical details of the CellSearch^® ^technique have been described elsewhere [11].

### Objectives and statistics

The study was powered for its main objective that consisted of detecting among patients with ≥ 5 CTC/7.5 ml at baseline, a 35% difference in six-month PFS rates between patients with a CTC count < or ≥ 5 CTC/7.5 ml before C2. The study was not specially powered for its planned secondary objectives, including this comparison with serum tumor markers. Data are expressed as means or numbers (%). Categorical variables were compared by chi-square or Fisher's exact test, and continuous variables were compared by Student's *t*-test or Wilcoxon's rank-sum test. Kaplan-Meier progression free survival (PFS) curves were computed according to CTC counts (< or ≥ 5 CTC/7.5 ml) and other tumor markers (< or ≥ Upper Limit of Normal Value (ULNV)), and were compared using log-rank tests. Progression was defined as local or distant relapse or death by any cause. Concordance index was also computed for each marker as continuous variables, PFS as outcome. Univariate analysis was performed using hazard ratios and 95% CI for PFS for each clinical characteristic and for each tumor marker or CTCs separately. A concordance index for PFS based on a clinical model (a model including all the above clinical characteristics) was used to show by how much each tumor marker or CTCs improved the performance of the clinical model in a multivariate analysis. Significance was defined as *P *≤ 0.05. Statistical analyses were performed using R2.12.1 software (Wien University, Wien, Austria)[12]

## Results

From June 2007 to September 2009, 267 patients with a median age of 57 years were included in the IC 2006-04 study. With a median follow-up of 14.9 months, 161 tumor progressions (60%) were recorded at the time of analysis.

### Detection rates at baseline

**Figure 1:**
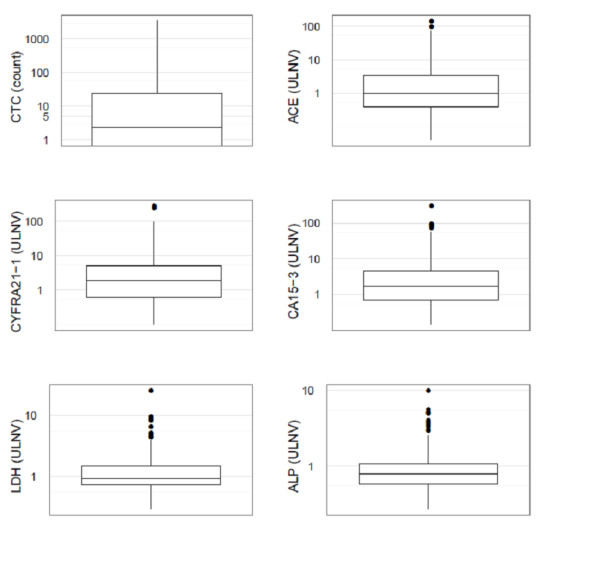
**Boxplot of CTC and serum markers values (semi log scale), median and CI 95%**.

CTC and serum marker values at inclusion repartition in percentile, mean, median range are given in Table 1 and Figure 1. Values for serum markers are expressed in ULNV: upper limit of normal value. Table 2 shows elevated serum marker (> ULNV) and CTC (≥ 5 CTC/ml) incidence rates at baseline: CA 15-3 and CYFRA 21-1 were the two most commonly elevated serum markers. Serum markers and CTC were highly correlated to performance status, number of metastatic sites and to each other. Table 3 shows the percentage of patients who had at least one marker elevated at baseline according to different marker combinations. As expected, this percentage globally increases with the number of markers assessed. However, the combination of CA 15-3 and CYFRA 21-1 retrieved almost the same positivity rate (86%) than all four markers (90%).

**Table 1:** CTC and serum marker values repartition at inclusion

	Mean	SD	Quantile 0%	Quantile 25%	Quantile 50% (median)	Quantile 75%	Quantile 100%	N
CTC	81.65	324.76	0	0	2.5	23.25	3,369	260
CA15.3	7.53	23.77	0.14	0.7	1.76	4.45	314.1	247
CEA	7.20	18.23	0.04	0.4	1	3.45	146.13	212
CYFRA21	9.01	29.51	0.1	0.65	1.95	5.25	284.54	191
LDH	1.39	2.02	0.28	0.71	0.92	1.45	25.54	220
ALP	1.056	1.00	0.26	0.58	0.79	1.06	10	241

CTC and serum markers values at inclusion repartition in percentile, mean, median range. Values for serum marker are expressed in ULNV, upper limit of the normal value

**Table 2:** Detection rates of elevated markers at baseline

Patient characteristics	N patients assessed	CA 15-3 > ULNV	CEA > ULNV	CYFRA 21-1 > ULNV	LDH > ULNV	ALP > ULNV	CTC ≥ 5
**Menopausal status**		NS	NS	***P *= 0.01**	NS	NS	NS
**Premenopausal**	113	42%	44%	**54%**	44%	25%	45%
**Postmenopausal**	148	58%	56%	**72%**	45%	33%	43%
**Receptor status**		NS	***P *= 0.002**	NS	NS	NS	NS
**Horm. positive**	159	68%	**58%**	65%	40%	33%	46%
**HER2 positive**	45	60%	**51%**	56%	49%	32%	34%
**Triple negative**	54	57%	**27%**	74%	53%	19%	46%
**Tumor grade**		NS	***P *= 0.05**	NS	NS	***P *= 0.03**	NS
**1**	26	77%	**76%**	64%	37%	**52%**	48%
**2**	106	64%	**55%**	62%	43%	**28%**	41%
**3**	123	63%	**45%**	68%	47%	**25%**	45%
**Performance status**		***P *= 0.003**	***P *= 0.02**	***P *< 10**^**-4**^	***P *< 10**^**-4**^	***P *= 0.001**	***P *< 10**^**-4**^
**0**	123	**55%**	**42%**	**51%**	**21%**	**18%**	**29%**
**1**	97	**69%**	**57%**	**70%**	**58%**	**34%**	**49%**
**2**	22	**82%**	**53%**	**79%**	**79%**	**52%**	**71%**
**3 or 4**	13	**92%**	**78%**	**100%**	**100%**	**54%**	**92%**
**Number of metastatic sites**		***P *= 0.02**	NS	***P *= 0.001**	***P *< 10**^**-4**^	NS	***P *= 0.03**
**< 3**	155	**60%**	52%	**55%**	**37%**	28%	**39%**
**≥ 3**	106	**71%**	51%	**80%**	**56%**	31%	**48%**
**CA 15.3**		-	***P *< 10**^**-4**^	***P *< 10**^**-4**^	***P *< 10**^**-4**^	***P *< 10**^**-4**^	***P *< 10**^**-4**^
**≤ ULNV**	88	-	**24%**	**54%**	**25%**	**13%**	**26%**
**> ULNV**	159	-	**68%**	**72%**	**56%**	**40%**	**53%**
**CEA**		-	-	***P *= 0.005**	***P *= 0.009**	***P *= 0.001**	***P *= 0.03**
**≤ ULNV**	103	-	-	**56%**	**36%**	**18%**	**35%**
**> ULNV**	109	-	-	**75%**	**55%**	**38%**	**49%**
**CYFRA 21-1**		-	-	-	***P *< 10**^**-4**^	NS	***P *< 10**^**-4**^
**≤ ULNV**	66	-	-	-	**16%**	23%	**19%**
**> ULNV**	125	-	-	-	**63%**	34%	**56%**
**LDH**		-	-	-	-	***P *= 0.02**	***P *< 10**^**-4**^
**≤ ULNV**	121	-	-	-	-	**22%**	**25%**
**> ULNV**	99	-	-	-	-	**36%**	**67%**
**ALP**		-	-	-	-	-	***P *< 10**^**-4**^
**≤ ULNV**	170	-	-	-	-	-	**36%**
**> ULNV**	71	-	-	-	-	-	**65%**
**All population** **at baseline**		64%	51%	65%	45%	29%	44%
**All population** **before cycle 2**		66%	46%	34%	43%	28%	17%
**All population** **before cycle 3/4**		64%	40%	27%	49%	22%	13%

ULNV: upper limit of normal value. *P*-values were obtained by Pearson's Chi-square test. Horm. positive: either estrogen and/or progesterone receptor positive by immunohistochemistry on primary tumor. HER2 positive: HER2 overexpressed by immunohistochemistry or amplified by FISH in 2+ cases. Triple negative: estrogen, progesterone and HER2 negative primary tumor. NS, non significant. Significant associations (*P *≤ 0.05) are in bold.

**Table 3:** Percentage of patients with at least one elevated marker at baseline

Markers assessed				N patients assessed	% with at least 1 elevated marker
**CEA**	**CA 15-3**	**CYFRA 21-1**	**CTC**		
**2 markers combinations**					
**X**			X	239	72%
**X**	X			240	75%
	X		X	257	75%
		X	X	225	77%
**X**		X		206	80%
	X	X		231	86%
**3 markers combinations**					
**X**	X		X	251	83%
**X**		X	X	233	86%
**X**	X	X		234	88%
	X	X	X	234	88%
**4 markers combination**					
**X**	X	X	X	246	90%

Single marker positivity rates are given for each of the markers, Table 2.

### Single assessment and association with PFS

**Figure 2:**
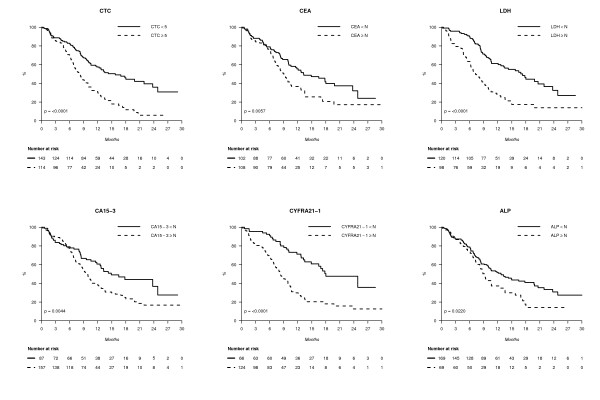
**Progression-free survival (PFS) according to blood markers at diagnosis**.

**Figure 3:**
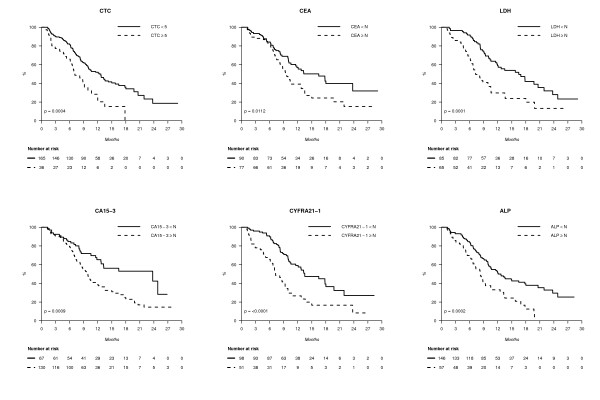
**PFS according to blood markers before cycle 2**.

When elevated, all markers tested at baseline had a negative prognostic impact in univariate analysis, as shown in Figure 2. The concordance index (and 95% confidence interval), which quantifies the quality of ranking and is a common performance measure for assessing learned models in survival analysis, was calculated for each marker, with PFS as the outcome. Concordance indexes with PFS were, at baseline, C-index_ALP _= 0.56 (0.50 to 0.61) (*n *= 238), C-index_CA15-3 _= 0.56 (0.51 to 0.61) (*n *= 244), C-index_CEA _= 0.58 (0.52 to 0.64) (*n *= 210), C-index_CTC _= 0.61 (0.56 to 0.66) (*n *= 257), C-index_CYFRA21-1 _= 0.67 (0.62 to 0.72) (*n *= 190) and C-index_LDH _= 0.68 (0.63 to 0.73) (*n *= 218). These results showed that the markers' performances were globally close to each other, with ALP having the worst result and CYFRA 21-1 and LDH the best. Similar results were obtained when markers were assessed during treatment, before cycle 2 (three to four weeks later, Figure 3) and before cycles 3 to 4 (six to nine weeks later, not shown).

In a univariate analysis for PFS, performance status, number of metastatic sites and triple-negative status were statistically significant for PFS (Table 4). At multivariate analysis, only performance status and triple negative tumor status were independent clinical prognostic factors and were included in a clinical model. CTC, LDH, CYFRA21.1, CEA and CA15.3 were statistically significant for PFS and not ALP. When the concordance index was calculated for each tumor marker or CTCs added to the clinical model, all of them, except ALP, slightly improved the performance of the clinical model. The C-index of the clinical model (0.697) increased from 0.71 to 0.723, except with ALP.

**Table 4:** Contribution of each serum marker and CTCs to a clinical model

Variable	Univariate	Multiv clin	Multiv clin + CTC	Multiv clin + CA 15.3	Multiv clin + CYFRA 21	Multiv clin + CEA	Multiv clin + LDH	Multiv clin + ALP
**Triple neg**.	3.05 (2.14; 4.36)	3.55 (2.43; 5.18)	3.33 (2.06; 5.39)	3.27 (2.03; 5.30)	3.14 (1.95; 5.06)	4.417 (2.628; 7.425)	3.061 (1.89; 4.95)	3.344 (2.062; 5.423)
**PS > 0**	2.30 (1.65; 3.21)	2.659 (1.89; 3.741)	2.097 (1.319; 3.33)	2.359 (1.509; 3.69)	2.133 (1.35; 3.36)	2.428 (1.56; 3.78)	1.968 (1.21; 3.19)	2.49 (1.58; 3.93)
**Nb of metastatic sites > 2**	1.886 (1.378; 2.581)	NS						
**CTC ≥ 5**	2.263 (1.644; 3.115)		1.996 (1.277; 3.122)					
**CA15-3 ≥ ULNV**	1.665 (1.167; 2.374)			1.746 (1.095; 2.783)				
**CYFRA 21-1 ≥ ULNV**	2.901 (1.896; 4.439)				2.097 (1.27; 3.46)			
**CEA ≥ ULNV**	1.64 (1.151; 2.337)					2.015 (1.272; 3.191)		
**LDH ≥ ULNV**	2.525 (1.781; 3.579)						1.933 (1.203; 3.104)	
**ALP ≥ ULNV**	1.501 (1.058; 2.131)							1.108 (0.684; 1.793)
**C-INDEX**		0.697 (0.65; 0.75)	0.71 (0.663; 0.767)	0.713 (0.661; 0.765)	0.723 (0.68; 0.77)	0.714 (0.664; 0.765)	0.717 (0.668; 0.766)	0.688 (0.637; 0.74)

Univariate analysis using hazard ratios and 95% CI for PFS for each clinical characteristic in the first column. Multivariate analysis for clinical parameters (second column) (Multiv. clin) Contribution for each tumor marker or CTCs to clinical model (Multiv.clin + serum marker) Last line: Concordance indexes for PFS for clinical model including all the clinical characteristics and for each tumor marker or CTCs.

### Changes under treatment and association with PFS

**Figure 4:**
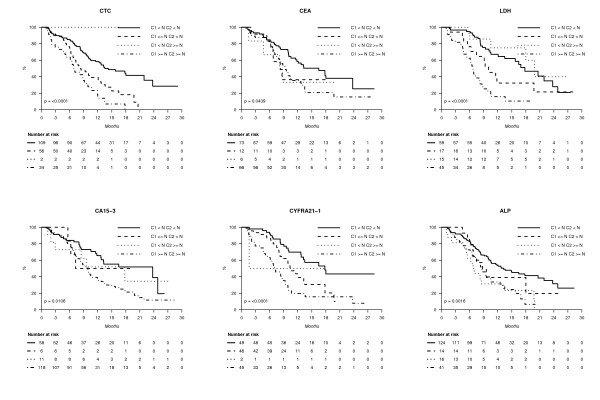
**PFS and marker changes between baseline and cycle 2**.

By combining marker status (elevated or not) before cycle 1 and before cycle 2, four subgroups could be separated for each marker. Figure 4 shows that these early changes were highly correlated with PFS in univariate analysis for all the markers; globally, patients with non-elevated markers at both time points had longer PFS; patients with elevated markers at both time points had generally the worst PFS. However, even in these groups of worst prognosis, median PFS was longer than six months for every marker tested, suggesting that none of these marker's early changes can predict accurately very short PFS. Concordance indexes with PFS were C-index_CTC _= 0.57 (0.51 to 0.63) (*n *= 201), C-index_ALP _= 0.50 (0.44 to 0.56) (*n *= 195), C-index_CEA _= 0.45 (0.38 to 0.51) (*n *= 157), C-index_CA15-3 _= 0.50 (0.44 to 0.56) (*n *= 193), C-index_CYFRA21-1 _= 0.57 (0.50 to 0.65) (*n *= 142) and C-index_LDH _= 0.63 (0.55 to 0.70) (*n *= 136). Again, these results showed that marker performances were extremely close to each other.

**Figure 5:**
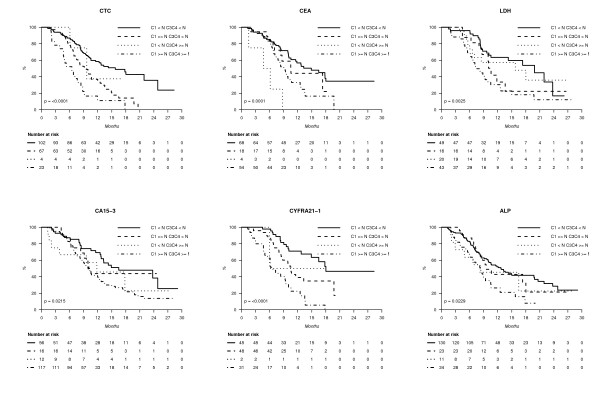
**PFS and marker changes between baseline and cycles 3 to 4**.

As a spurious early rise of serum markers may occur during the first weeks of a new chemotherapy and may lower the above serum markers' concordance indexes, we also studied the changes that occur before cycle 1 and before cycles 3 to 4; Figure 5 shows that these changes were also significant for all the markers. We calculated the concordance indexes by combining marker status before cycle 1 and before cycle 3/4 (weeks 6 to 9). The concordance indexes for the five serum markers and for CTC were all between 0.61 and 0.66, with largely overlapping 95% confidence intervals.

### CTC prognostic value according to serum marker subgroups

Finally, interaction tests have been performed to further study if the prognostic value of baseline CTC count and of CTC count changes under treatment were restricted and/or different according to the different patient's subgroup, including serum marker subgroups (for example, in patients with non-elevated CEA *vs *elevated CEA). These tests were non significant.

## Discussion

The need for novel independent prognostic factors in metastatic breast cancer patients is much lower than the need for dynamic blood markers, which can indicate the treatment efficiency in a reliable and early fashion. Serum tumor markers are an easy, quick, cheap, but rather imprecise and sometimes misleading tool, to monitor the treatment efficacy. However, they are particularly valuable for treatment monitoring in patients that have disease that cannot be evaluated by radiology. CTC count by the CellSearch^® ^system is a validated prognostic factor at baseline, but is also used for treatment monitoring [13-15]. This test is, however, more expensive than serum tumor markers and has never been directly compared with them. The IC 2006-04 is the first large prospective study in which a comparison between CTC and five different serum markers was pre-planned.

At first, our study tried to answer a recurrent issue for medical oncologists, that is "which serum marker should I ask for?" To be considered as informative and usable for further determination under therapy, a serum marker should be elevated at baseline, before the start of therapy. Historically, comparison between serum markers mostly used positivity rates (> ULNV) at baseline in metastatic patients [16]. For serum markers and CTC, incidence rates were in line with those previously reported at first metastatic relapse [17,18], together with the low incidence of CEA in triple-negative cancers [19]. Our study shows that repeated CTC counts are informative mainly for patients who have an elevated level at baseline (≥ 5CTC/7.5 ml), but not for patients initially CTC negative (< 5CTC/7.5 ml), as only two of these patients will present an increase (≥ 5CTC/7.5 ml) during treatment (Figure 3). Due to the very small number of these patients, no conclusion can be drawn on the prognostics of this subgroup. On the basis of baseline positivity rate comparison, assessing only CEA and CA 15-3, the two most commonly used breast cancer serum markers retrieve around 75% of patients with at least one marker elevated at baseline. Our study has also investigated CYFRA 21-1 as a breast cancer serum marker, which turned out to be the most commonly elevated serum tumor marker (65% of patients), as already suggested in smaller studies [20,21]. Adding either CYFRA 21-1 or CTC to this CEA and CA 15-3 combination further increases this percentage to around 90% of patients, but assessing the full four-marker panel (CEA, CA 15-3, CYFRA 21-1 and CTC) did not further improve the overall positivity rate. Among the different markers tested, a drop in positivity rates between baseline and cycle 3/4 was mostly observed for CTC and CYFRA 21-1: 44 to 13% and 65 to 27% respectively (Table 2).

We showed that serum markers and CTC positivity were highly correlated with other known clinical prognostic factors, such as poor performance status or the high number of metastatic sites. Interestingly, neither serum markers nor CTC detection were correlated with immunohistological subtypes (hormone receptors-positive, HER2-positive or triple-negative breast cancers). Unsurprisingly, each of the markers tested had a significant impact on PFS in univariate analysis. Multivariate analyses, including serum markers, CTC and known clinical prognostic factors, have been reported previously: CTC and CEA were the two blood markers independently associated with PFS, whereas CTC count was the only blood marker independently associated with OS [9].

Here, by comparing the early and late changes of five blood markers together with CTC changes for PFS prediction, we showed no clear superiority of CTC over the other serum markers. This result was, however, not the primary endpoint of our study, and the statistical power of these analyses may still be discussed, although performed in more than 200 patients. For this analysis, we used the "prognosis-optimized" threshold of ≥ 5 CTC/7.5 ml, which was initially defined as the best dichotomizing threshold for PFS and OS prediction by CTC at baseline and under treatment [7]. Contrarily, for serum tumor markers, the upper limits of normal values (ULNV) were historically defined by healthy-donor analyses, and no cut-off has been optimized previously for survival prediction. In this study, it was pre-analytically planned to use ULNV as the positivity threshold at both baseline and before cycle 2 and, as for CTC counts, to divide the population into four subgroups according to the marker at baseline and before cycle 2. Other models may have been used for serum markers, in order to take into account the relative changes of elevated marker (for example, 50% decrease between baseline at cycle 2...), but testing all the possibilities for serum marker analyses would have led to multiple testing biases favoring serum markers over CTC.

## Conclusion

Finally, the IC 2006-04 study suggests that CTC count should not be considered alone, but should be put into the current clinical context of metastatic breast cancer. We previously demonstrated that CTC, but not serum markers, has an independent prognostic value in multivariate analysis. This prognostic value did not interact with any tested subgroup. However, when focusing on the different blood markers assessable for PFS prediction before and during treatment, we report here that there is no clear superiority of CTC count over serum markers. Markers predicting or monitoring treatment efficiency are much more needed for patients with metastatic breast cancer than prognostic serum markers. A further prospective comparative study would be required to obtain a definite conclusion with a predefined statistical power. As, to our knowledge, no such study is ongoing, we believe that the IC 2006-04 will remain the largest prospective study with a CTC/serum marker comparison. The interest of implementing CTC count into the routine management of metastatic breast cancer chemotherapy will be finally assessed in the two ongoing randomized trials SWOG500 (USA) and CirCe01 (France).

## Abbreviations

ALP: alkaline phosphatase; C2: cycle 2 (of chemotherapy); C3/4: cycle 3/4 (of chemotherapy); CA: cancer antigen; CEA: carcinoembryonic antigen; CTC: circulating tumor cell; LDH: lactate deshydrogenase; OS: overall survival; PFS: progression-free survival; ULNV: upper limit of normal value.

## Competing interests

J-YP received research grants and lecture honoraria from Veridex. F-CB received lecture honoraria from Veridex. The other authors have no disclosures. This study was presented in part at the 2010 San Antonio Breast Cancer Symposium and at the 2011 IMPAKT conference (oral presentations).

## Authors' contributions

FCB participated to data collection, data interpretation and statistical analyses, and wrote the manuscript. DH supervised data management and performed statistical analyses. TB, SD, EB, MC, PC and PB participated in the design of the study and included patients in the study. ER coordinated data management and participated in statistical analyses. CM supervised the central laboratory for CTC detection. JYP conceived of the study, and participated in its design and coordination, and data interpretation and manuscript writing. All authors read and approved the final manuscript.
